# Use of the WISN method to assess the health workforce requirements for the high-volume clinical biochemical laboratories

**DOI:** 10.1186/s12960-021-00686-w

**Published:** 2022-01-28

**Authors:** Sanja Stankovic, Milena Santric Milicevic

**Affiliations:** 1 Center for Medical Biochemistry, University Clinical Center of Serbia, Visegradska 26, 11000 Belgrade, Serbia; 2 Faculty of Medical Sciences, University of Kragujevac, Svetozara Markovica 69, 34000 Kragujevac, Serbia; 3 Institute of Social Medicine, Faculty of Medicine, University of Belgrade, Dr Subotica 15, 11000 Belgrade, Serbia

**Keywords:** Laboratory, Human resources management, WISN method, Activity standards, Planning, Staffing, Workload indicators

## Abstract

**Background:**

The clinical laboratory services, as an essential part of health care, require appropriate staff capacity to assure satisfaction and improve outcomes for both patients and clinical staff. This study aimed to apply the Workload Indicators of Staffing Need (WISN) method for estimating required laboratory staff requirements for the high-volume clinical biochemical laboratories.

**Methods:**

In 2019, we applied the WISN method in all 13 laboratories within the Center for Medical Biochemistry of the University Clinical Centre of Serbia (CMB UCCS). A review of annual routinely collected statistics, laboratory processes observations, and structured interviews with lab staff helped identify their health service and additional activities and duration of these activities. The study outcomes were WISN-based staff requirements, WISN ratio and difference, and a recommendation on the new staffing standards for two priority laboratory workers (medical biochemists and medical laboratory technicians).

**Results:**

Medical biochemists’ and laboratory technicians’ annual available working time in 2019 was 1508 and 1347 working hours, respectively, for the workload of 1,848,889 samples. In general, the staff has four health service, eight support, and 15 additional individual activities***.*** Health service activities per sample can take from 1.2 to 12.6 min. Medical biochemists and medical laboratory technicians spend almost 70% and more than 80% of their available working time, undertaking health service activities. The WISN method revealed laboratory workforce shortages in the CMB (i.e. current 40 medical biochemists and 180 medical laboratory technicians as opposed to required 48 medical biochemists and 206 medical laboratory technicians). Workforce maldistribution regarding the laboratory workload contributes to a moderate–high workload pressure of medical biochemists in five and medical laboratory technicians in nine organizational units.

**Conclusions:**

The WISN method showed mainly a laboratory workforce shortages and workload pressure in the CMB UCCS. WISN is a simple, easy-to-use method that can help decision-makers and policymakers prioritize the recruitment and equitable allocation of laboratory workers, optimize their utilization, and develop normative guidelines in the field of clinical laboratory diagnostics. WISN estimates require periodic reviews.

**Supplementary Information:**

The online version contains supplementary material available at 10.1186/s12960-021-00686-w.

## Background

Timely and accurate laboratory test results are an integral part of many clinical decisions about prevention, diagnosis, treatment choice, and health and disease management [1]. Medical laboratory professionals are one of the broad categories of the health workforce [2]. Medical laboratories have been afflicted with shortages and skill-mix adequacy of laboratory professionals for many decades [3]. Still, their capacity and equitable distribution have been a rare focus of the research for many decades despite the workplace’s technology progress [4, 5] before the COVID-19 pandemic, during which rapid and accurate testing of large number of samples is necessary. For the second year in a row with the COVID-19 pandemic, laboratory managers as transformational leaders are trying to meet the growing demand for services within limited laboratory budgets, mainly by improving productivity and clinical excellence (e.g., shortening turnaround times, using advanced technologies, maintaining service quality and safety, and customer satisfaction) [6].

Laboratory managers have long struggled with staffing formula due to the complex nature of laboratory activities [7]. In many countries, they traditionally rely on simple methods for determining the staff number and composition using the density rate (i.e. the ratio of staff to population method). In Serbia, the laboratory staffing is based on the current regulations of the Republic of Serbia [8, 9], which determine the norm for the number of necessary health workers in laboratory diagnostics on the scope of work of 120,000 tests per year (that is, one medical biochemist and six medical laboratory technicians per 120,000 tests per year). The use of uniform staffing standards regardless of the type of laboratory work can create real problems in providing some laboratory services, as laboratories vary widely in many respects, including the level of healthcare in which they are integrated, their size, complexity of operations, working hours, laboratory staff qualifications, and activities.

From the late 1990s, the World Health Organization (WHO) provided the Workload Indicator of Staffing Need (WISN) method to be used for estimating the number of staffing needs based on the actual workload and forecasting the human resources required for different clinical settings in numerous countries [10]. The implementation of WHO/WISN method has been mainly explored for nurses, midwifes and physicians [11–13], while only a few studies have targeted laboratory staff [14–17]. Research on the application of the WISN method in high-volume medical laboratories could be a valuable contribution to the scarce literature on medical laboratory workers. This study aims to assess the staff requirements according to the workload in high-volume clinical biochemical laboratories, which will provide evidence to improve their equitable distribution and management at the institutional level.

## Methods

We have applied the WISN methods in the Center for Medical Biochemistry of the University Clinical Centre of Serbia (CMB UCCS) in 2019. CMB UCCS is the leading reference institution in Serbia in laboratory diagnostics (clinical chemistry, immunochemistry, immunology, haematology/haemostasis, electrophoresis, chromatography, non-standard fluid analysis, on-site testing, blood gas analysis, etc.). The study population consists of 220 health laboratory workers full-time equivalents (FTEs), including medical biochemists and medical laboratory technicians employed in all 13 CMB UCCS laboratories, who were functionally organized to provide services in 13 separate UCCS clinics (Additional file 1). 

The study followed the steps of the WHO/WISN Manual [10] to calculate staff requirements and assess the workload pressure of the laboratory workers in the CMB facility. Data sources were semi-structured interviews with key personnel of all categories of staff, observation of the work processes (Additional file 2), laboratory records and regulations containing job descriptions and the standard operating procedures.

Furthermore, we applied the WISN findings to assess the CMB UCCS staffing required to perform 120,000 tests, performance measure defined by national bylaws [8, 9]. In addition, we calculated the staff rate per 100,000 patient-population considering 887,888 patients covered by CMB services in 2019.

The study data were collected in a mixed-method approach. An overview of CMB UCCS annual personnel records provided information on laboratory staff number and FTE workers, age and sex structure, and professional category. An assessment of staff payments official records, taken directly from the institutional department of financial administration helped calculate of Available Working Time (AWT). By reviewing the regulatory framework (legislation, job descriptions, standard operating procedures, reporting schemes, etc.), we procured the information necessary to understand the functions and activities of the laboratory, standards of laboratory practice, and operating procedures. We have observed the laboratory processes’ phases 20 times in 5 days. During the observation study, we have measured the time necessary to perform every health services activity of the biochemist and medical laboratory technicians. To reduce variability of measurement duration, the average time of 100 observations for each activity performed by laboratory staff was taken as the unit time for each health service activity. A review of daily laboratory records and periodic and annual reports helped us capture the nature of the work of the 13 laboratories, including the daily scope and type of testing performed by workforce category. We interviewed key personnel of all categories of staff about all activities per sample from pre-analytic to post-analytic phase (presented in flowcharts in Additional file 3) that we previously observed in a time–motion study and record analysis. Next, we have presented our findings to the interviewing staff, to be able to jointly set the respective activity standard.

The laboratory managers and staff decided to present workload as samples (i.e. patients’ specimens). The number of samples is routinely collected in laboratory records and laboratory information system (LIS) and registered in regular statistics consisting of official quarterly, semi-annual, and annual statistical reports of the laboratory. The annual records for the 3-year reporting period, i.e. from 2017 to 2019, showed that the annual workload was similar during that period, and it changed ± 0.06%. 

Using regular statistics for 2019, we also calculated the percentage of different samples with regard to total sample size for each of the 13 laboratories in order to see the laboratory workload structure per sample type (i.e. serum samples used for clinical chemistry/immunochemistry test determination, plasma samples for coagulation tests, plasma samples for erythrocyte sedimentation rate, whole blood for complete blood count with differential, urine samples, feces, semen, and cerebrospinal fluid). The time needed for active engagement of a medical laboratory technician in analytical phase of every sample type was measured 20 times in 5 days (i.e. 100 observations for each activity was performed). Finally, we calculated the average time for analysis of all samples in each laboratory.

The study was approved by the Ethical Board of Clinical Centre of Serbia (Permission No: 692/10 from May 17, 2018). The informed consent of interviewed personnel was recorded on the condition of strict respect for privacy and confidentiality.

## Results

CMB UCCS is the first clinical laboratory in Serbia to introduce a quality management system according to the requirements of ISO 9001 in 2000, ISO 17025 in 2006, ISO 15189 in 2008 (flex scope of accreditation from 2013), and since 2016, the Accreditation Agency of Health Institutions of Serbia has accredited the CMB UCCS. It is the teaching base for university students and specialists in medical/clinical biochemistry. CMB UCCS is a partner in the Republic Expert Commission for Medical and Clinical Biochemistry activities and research projects.

### The priority cadres for WISN analysis, available working time, and workload components

The study population is 220 FTE health laboratory workers, including 40 FTE medical biochemists and 180 FTE medical laboratory technicians. Laboratory workers are mostly females (88.9% of medical biochemists and 81.8% medical laboratory technicians). The median age of medical biochemists and laboratory technicians is 50 years. Medical biochemists have various university-level degrees (bachelor’s degrees in pharmacy, master’s degrees in medical biochemistry, Ph.D. in medical biochemistry, specialization in medical biochemistry, and subspecialization in clinical enzymology, clinical endocrinology, immunochemistry). Medical laboratory technicians (and technologists) have secondary or college medical education.

In 2019, the AWT for a medical biochemist of CMB UCCS was 225 days, that is calculated as the difference from the total of 308 working days (365 days in a year including lieu days and excluding free weekends) and 83 days-off (including an average of 36 days of annual leave, 5 days for sick leave, 5 days for maternity leave and 37 days for other reasons, such as holidays, days-off after duty, etc.). Using the same formula, the AWT for medical laboratory technicians was 201 days (266 working days and 65 days off (including an average of 35 days of annual leave, 13 days for sick leave, 8 days for maternity leave, and 9 days for other reasons). Line managers verify all grounds for leaves (e.g., free days, holidays, training sessions, etc.). Managers reported that medical laboratory technicians take more maternity leaves than medical biochemists because there are of reproductive age and have smaller children who are often ill and use sick leave for that reason. In case of absence, other staffs work instead of laboratory technicians (this is not “work on-call”). 

In CMB UCCS, a laboratory worker has 36 working hours per week [8]. The number of working hours a day was 6.7 h, after excluding half an hour for a break, as per bylaw [9]. Therefore, in 2019, the estimated AWT was 1508 h (90,480 min) for a medical biochemist and 1347 h (80,820 min) for a medical laboratory technician in CMB UCCS.

For all 13 separate CMB UCCS laboratories, medical biochemist’ workload components include health service activities (grouped in the post-analytic phase), four support categorical activities, and eight additional individual activities. Similarly, medical laboratory technicians have three health service activities (grouped in the pre-analytic, analytic, and post-analytic phase), four additional categorical activities, and seven additional individual activities.

Health service activities for medical biochemists comprise the following activities in the post-analytical phase (verification laboratory results and release of the laboratory test report per sample). Health service activities for medical laboratory technicians comprise the following activities on the sample in the pre-analytical phase (sample reception and registration, sampling, labelling, triage, putting it on centrifuge/removing from centrifuge, de-capping), in the analytical phase (putting the sample on pre-analytical system or putting it on analyser) and in the post-analytical phase (entering test results into the laboratory protocol and laboratory report (in laboratories without LIS), removing the sample from core analyser, putting it on storage racks, archiving storage racks in refrigerators, packing sample tubes in yellow clinical waste bags for waste disposal, and putting bags in large yellow clinical waste bin for waste disposal outside the laboratories.

Support categorical activities for medical biochemists and medical laboratory technicians and actual working time are shown in Additional file 4: Tables S1A and S1B), respectively.

Additional file 4: Tables S2A and S2B present the additional individual activities for medical biochemists and medical laboratory technicians, respectively, and the number and the time duration of each activity.

### Annual workload, activity standards, standard annual workload and allowance factors

In 2019, CMB UCCS provided services for 887,888 patients and performed about 12,289,890 tests for 1,848,889 samples (Table 1A, B). Medical biochemists and laboratory technicians spend almost 80% and 70% of their AWT, respectively, in performing health service laboratory activities per sample, and for the remaining time carry out additional category and individual activities (Table 1A, B).

**Table 1:** Annual workload, activity standards and total required number of medical biochemists (A)/medical laboratory technicians (B( based on WISN for 13 laboratories, Center for Medical Biochemistry University Clinical Center of Serbia, 2019

CMB UCCS laboratories	Annual workload (number of samples)	Activity standards			Total required staff for health service activities (*A*)	Category allowance factor (CAF) (*B*)	Individual allowance factor (IAF) (*C*)	Total required number of staff based on WISN (*A* × *B* + *C*)
		Health service activities, (minutes per sample)	Category additional activities (CAS) (% AWT)	Individual additional activities (IAS) (hours per year)				
A								
Total	1,848,889							
1PD	387,277	1.2	16.26	5305.2	5.14	1.19	3.52	9.64
2ED	707,961	1.2	16.26	5466.0	9.39	1.19	3.62	14.79
3INF	79,217	1.2	16.26	764.2	1.05	1.19	0.51	1.76
4GO	82,188	1.2	16.26	651.7	1.09	1.19	0.43	1.73
5CS	157,207	1.2	16.26	764.2	2.08	1.19	0.51	2.99
6NS	71,263	2.0	16.26	651.7	1.58	1.19	0.43	2.31
7E	126,141	1.2	16.26	651.7	1.67	1.19	0.43	2.42
8H	38,963	1.2	16.26	764.2	0.52	1.19	0.51	1.13
9U	25,450	1.2	16.26	651.7	0.34	1.19	0.43	0.83
10DS	91,145	2.0	16.26	651.7	2.01	1.19	0.43	2.82
11N	38,350	2.0	16.26	764.2	0.85	1.19	0.51	1.52
12O	37,150	1.2	16.26	651.7	0.49	1.19	0.43	1.01
13P	6577	2.0	16.26	651.7	0.15	1.19	0.43	0.61
B								
Total	1,848,889							
1PD	387,277	8.0	26.87	4508.4	24.14	1.37	3.35	36.42
2ED	707,961	10.3	26.87	1560.1	46.07	1.37	1.16	64.28
3INF	79,217	8.8	26.87	694.7	7.65	1.37	0.52	11.00
4GO	82,188	9.2	26.87	592.2	8.79	1.37	0.44	12.48
5CS	157,207	6.3	26.87	593.2	12.25	1.37	0.44	17.22
6NS	71,263	8.9	26.87	592.2	7.85	1.37	0.44	11.19
7E	126,141	6.6	26.87	593.2	10.30	1.37	0.44	14.55
8H	38,963	8.1	26.87	592.2	3.90	1.37	0.44	5.78
9U	25,450	11.6	26.87	593.2	3.65	1.37	0.44	5.44
10DS	91,145	12.6	26.87	593.2	9.55	1.37	0.44	13.52
11N	38,350	8.9	26.87	592.2	4.22	1.37	0.44	6.22
12O	37,150	6.8	26.87	592.2	3.13	1.37	0.44	4.73
13P	6577	8.5	26.87	592.2	0.69	1.37	0.44	1.39

Division of Polyclinic Laboratory Diagnostics (1PD), Division of Emergency Laboratory Diagnostics (2ED), and Division of Clinical Laboratory Diagnostics in Department in the Clinic for Infectious and Tropical Diseases (3INF), Department in the Clinic for Gynecology and Obstetrics (4GO), Department in the Clinic for Cardiac Surgery (5CS), Department in the Clinic for Neurosurgery (6NS), Department in the Clinic for Endocrinology, Diabetes and Metabolic Diseases (7E), Department in the Clinic for Hematology (8H), Department in the Clinic for Urology (9U), Department in the Clinic for Digestive Surgery (10DS), Department in the Clinic for Neurology (11N), Department in the Clinic for Orthopedics and Traumatology (12O), Department in the Clinic for Burns, Plastic and Reconstructive Surgery (13P)

Activity standards for health service activities per sample ranged 1.2–2.0 min for medical biochemists (Table 1A) and 6.3–12.6 min for medical laboratory technicians (Table 1B). The standard annual workload for medical biochemist’s health service activities has ranged from 3289 samples to 589,968 samples. The standard annual workload for health service activities of medical laboratory technicians has varied among labs in the range from 774 samples to 68,734 samples.

Support categorical activities for medical biochemists and medical laboratory technicians take 16.26% and 26.87% of AWT, respectively. To perform additional individual activities, medical biochemists spend 651.2–5466.0 h per year, while medical laboratory technicians spend 592.2–4508.4 h per year, depending on the laboratory (Table 1A, B).

For medical biochemists, the CAF was 1.19 in all laboratories, while the minimum IAF was 0.43 and the maximum was 3.62 (Table 1A). For medical laboratory technicians, the CAF was 1.37 and IAF ranged from 0.44 to 3.35 (Table 1B).

### The staffing requirements, workload pressure, and staffing norms

In 2019, five CMB UCCS laboratories had a shortage of 20.0% of total FTE medical biochemists (i.e. 8 FTE medical biochemists or 12,064 work hours in a year) and a moderate–high workload pressure (Table 2). Nine CMB UCCS laboratories have a shortage of 14.4% of total FTE medical laboratory technicians (i.e. 26 FTE medical laboratory technicians or 35,022 work hours per year), and two have high and seven moderate workload pressure. On the other hand, one laboratory has a surplus of medical laboratory technicians.

**Table 2:** Actual stock and WISN estimates of the FTE laboratory staff in 13 laboratories, Center for Medical Biochemistry University Clinical Center of Serbia, 2019

CMB UCCS laboratories	Staff category: FTE Medical biochemists, *n*						Staff category: FTE Laboratory technicians, *n*					
	Actual staffs (1)	WISN based staff (2)	WISN difference (1–2)	Workforce problem	WISN ratio (1/2)	Workload pressure	Actual staffs (1)	WISN based staff (2)	WISN difference (1–2)	Workforce problem	WISN ratio (1/2)	Workload pressure
Total	40	48	− 8	Misbalance			180	203	− 26	Misbalance		
1PD	9	10	− 1	Shortage	0.9	Moderate	33	36	− 3	Shortage	0.9	Moderate
2ED	15	15	0	Balance	1.0	Normal	65	64	1	Balance	1.0	Normal
3INF	2	2	0	Balance	1.0	Normal	12	11	1	Surplus	1.1	Low
4GO	2	2	0	Balance	1.0	Normal	11	13	− 2	Shortage	0.8	Moderate
5CS	2	3	− 1	Shortage	0.7	Moderate	13	17	− 4	Shortage	0.8	Moderate
6NS	1	3	− 2	Shortage	0.3	High	9	11	− 2	Shortage	0.8	Moderate
7E	1	3	− 2	Shortage	0.3	High	11	15	− 4	Shortage	0.7	Moderate
8H	2	2	0	Balance	1.0	Low	6	6	0	Balance	1.0	Normal
9U	1	1	0	Balance	1.0	Normal	5	6	− 1	Shortage	0.8	Moderate
10DS	1	3	− 2	Shortage	0.3	High	6	14	− 8	Shortage	0.4	High
11 N	2	2	0	Balance	1.0	Normal	3	6	− 3	Shortage	0.5	High
12O	1	1	0	Balance	1.0	Normal	4	5	− 1	Shortage	0.8	Moderate
13P	1	1	0	Balance	1.0	Normal	2	2	0	Balance	1.0	Normal

Division of Polyclinic Laboratory Diagnostics (1PD), Division of Emergency Laboratory Diagnostics (2ED), and Division of Clinical Laboratory Diagnostics in Department in the Clinic for Infectious and Tropical Diseases (3INF), Department in the Clinic for Gynecology and Obstetrics (4GO), Department in the Clinic for Cardiac Surgery (5CS), Department in the Clinic for Neurosurgery (6NS), Department in the Clinic for Endocrinology, Diabetes and Metabolic Diseases (7E), Department in the Clinic for Hematology (8H), Department in the Clinic for Urology (9U), Department in the Clinic for Digestive Surgery (10DS), Department in the Clinic for Neurology (11N), Department in the Clinic for Orthopedics and Traumatology (12O), Department in the Clinic for Burns, Plastic and Reconstructive Surgery (13P)

Using WISN estimates, for the 2019 workload, the CMB UCCS requires 0.5 medical biochemist and 2 medical laboratory technicians to perform 120,000 laboratory tests, or 6 medical biochemists and 23 medical laboratory technicians per 100,000 patient-population.

## Discussion

This study, for the first time in our country, using WISN method in the ISO-accredited high-volume clinical laboratories of a tertiary care University Clinical Centre of Serbia, has estimated the optimal number of medical biochemists and medical laboratory technicians for the actual workload. The WISN difference indicates a shortage of 20% of FTE medical biochemists and 14% of FTE medical laboratory technicians and remarkably variable workload pressure (inequities in its distribution). Inequitable distribution of medical laboratory technicians is more serious than of medical biochemists across all 13 observed laboratories, as nine laboratories are in deficit of laboratory technicians while five laboratories have a deficit of medical biochemists. This shortage is equally pronounced in Serbian public health laboratories as well [18], and in other countries, which have analysed the staffing needs of laboratory technicians [14–17].

In the last decade, we are witnessing an explosion of new technologies and solutions in laboratories. However, largely outdated staffing norms leave the issue of inequitable distribution of laboratory workers largely unresolved. As the leader in laboratory diagnostics in Serbia, the CMB UCCS wanted to draw attention to the Ministry of Health Republic of Serbia to revise largely outdated staffing norms and develop an evidence-based staffing norm (staffing standard) using the WHO/WISN methods. In the absence of evidence-based guidelines, the standard operating procedures (SOPs) of laboratory processes are driven in part by regulations, by primary, secondary, and tertiary health care laboratory services, and by the priorities of the healthcare institutions. A person in charge of laboratory services in the UCCS has to identify which laboratory staff categories in which laboratories have the highest workload pressure and correct the misbalances. For that purpose, we have developed the project prospects, including the application of the WISN methods that has helped in identifying the activities of our laboratory staff and to streamline them. This was possible through direct identification of times spent on activities, and creation of a list of activity standards, which supported a systematic structuring of working schedules of the laboratory staff.

Due to advanced analytical technologies in medical laboratories, compared to the period of only 10 years ago [19, 20], the job description and laboratory workload, the structure of the services they provide, and the role of medical biochemists has changed a lot in Serbia as well. Today, a biochemist is more involved in post-analytical phase, including verification of laboratory results and release of the laboratory test report and communication with a physician/clinical department (unexpected test results, need for new sampling, reflex testing per sample, etc.) [21, 22]. Medical laboratory technicians are mainly involved in pre-analytical, analytical and partly in post-analytical phase of laboratory process, which is in line with the job descriptions of the Serbian laboratory workforce [8, 9].

The laboratory health sector of Serbia has over the years dealt with inadequate numbers and unbalanced skill-mix of the required laboratory workers as well as ill distribution. It was partly due to understaffing in some facilities and overstaffing in the others. Since 2014, only selective employment in the public sector, including health care, has been allowed, while the number of job posts is limited. However, even when the Ministry of Health of the Republic of Serbia approves new employments of medical laboratory technicians, an insufficient number of candidates apply for vacancies. The main reasons for unfilled vacancies and severe difficulties in recruitment efforts could be found in the work complexities, increased automation, and queries for higher salaries for the job of laboratory personnel.

The 2019, a job satisfaction survey [23] showed that 71.8% of CMB UCCS staff was dissatisfied with the salary and 42.3% with their performance evaluation, and 38.7% face work pressure, while 86.6% were satisfied with work equipment, 82.7% with cooperation with colleagues and 75.8% with superiors, and 77.5% with opportunities for further development/education.

Based on the WHO/WISN method for the 2019 workload, the study proposes new staffing norms in the highly automated medical biochemical laboratories at the tertiary health care level. Our results indicate that maintaining the quality of laboratory work and timely availability of their services with the current staffing is challenged. To balance the staffing to the present workload, these laboratories need an additional 8 FTE analysts and 26 FTE technicians. It is evident that in all organizational units, without laboratory information system and total laboratory automation, medical biochemists and medical laboratory technicians spend much more time on health service activities. However, the introduction of innovations in laboratory diagnostic field could concur with prolonged working time, stress, fatigue, resistance and absenteeism of an already overworked staff, and compromise quality of outcomes. Together with the redeployment of staff or workload to achieve WISN ratio balance, laboratory managers should effectively communicate WISN to gain staff motivation for workforce equity and should promote WISN benefits for CMB UCCS efficiency and quality as well as encourage staff role in upgrading/maintaining competencies.

This study is the first in Serbia that used the WISN to illustrate laboratory process organization, create a list of health services, support categorical and additional individual activities together with time necessary for their execution, and to develop staffing norms for medical biochemists and medical laboratory technicians in highly automated tertiary level health care laboratories. It provides evidence that could support the fact that the staffing norms cannot be equated for laboratories at the primary, secondary, and tertiary levels of health care, which have different workload, different levels of automation of the laboratory processes or without total automation, as well as with or without laboratory information system.

Laboratory staff shortage, aged pool of the personnel, and the demographic time bomb of looming retirements can only make the situation worse. The impact of inadequate laboratory staff becomes apparent when a delayed diagnosis or error occurs reducing the quality and optimal care of the patient [24–26]. The essential question of management is how to keep well-functioning and efficient medical laboratory organization capable of responding to workload and all challenges, especially to have cohesive and effective laboratory staff [27]. Therefore, the application of WISN analysis in CMB UCCS is particularly important for maintaining and improving the quality of health services and to improve patient safety and patient, physician, and employee satisfaction. On one hand, medical laboratories are under high pressure due to an increased number of patients, expanding test menus and increasing pressure to embrace the accelerated technological advancement such as an installation of total automation solutions and improvements in informatics accompanied by trainings and intensive additional activities. On the other hand, they are facing economic pressure to reduce costs, shortening turnaround time, and improve patient safety.

The study has both short-term and long-term recommendations. Based on the present study, in 2020, we have reorganized the work including the following:

- The relocation of a few laboratory workers from low to high workload laboratory unit;
- The shift of some current additional activities to other staff;
- Finishing the digital connection of laboratories to hospital information system;
- Hiring the additional workers on temporary base, and
- Recruiting more medical biochemists/laboratory technicians on mandatory internship.

However, due to the COVID-19 pandemic, a new laboratory department of UCCS is established in the newly built hospital for patients with COVID-19 near Belgrade, where the new laboratory staff was redirected, instead of employment in vacant positions in CMB UCCS.

In the long-run, this study recommendation is oriented toward policy and includes:

- Defining positions for separate staff category for administrative/support activities in laboratories;
- Improving laboratory staff education policy;
- Increase the number of residency training programs and positions especially at high-level laboratories;
- Revision of work-based staffing models to ensure adequate staff numbers and skills employed/maintained at the right time and place, and
- Development of strategies to recruit adequate numbers of laboratory staff.

Also, continuing with further application of the newest technology, total laboratory automation of our lab and application of different informatics solution (analyser management system, inventory management system, etc.) will ease workforce shortages, decrease the working time for some support and additional activities and enable delivery of greater overall productivity with their existing resources.

Adoption of flexible health workforce planning, and recruitment policy based on local patient load and diseases burden plausible future scenarios is highly desirable. More success is expected in workforce policy-making for CMB if these recommendations are in parallel with an integration of multi-site laboratories into one totally automate and fully digitalized laboratory facility. This 2019 study is a baseline study of the impact of the COVID-19 pandemic on the laboratory work during 2020 and 2021. It provides evidence to the Ministry of Health of the Republic of Serbia and UCCS Management’s for the reorganization of the UCCS laboratory service in 2021.

The adoption and application of the WHO/WISN methodology should be viewed as a vital tool in improving strategic health workforce planning and management in laboratory settings as well. In a broader context, the national health system can benefit from the use of the WHO/WISN method, not only in estimating the optimal number of laboratory staff and precisely defining the workload components, but also in revising of the staffing norms, improving staffing equity and productivity across the types of facilities, and estimating workforce requirements for new cadres in the near future.

### Limitations

A few limitations of our study need to be highlighted. Firstly, our survey has covered only the year 2019, and it is possible that attrition trends may vary over the years. Given that at the time of this study there was no digitized database of laboratory staff’s personnel information, data about annual leaves, sick leaves and other leaves were available only in paper form, and the process of obtaining this data was slow. However, to reduce the possible biases we have overviewed AWT and workload data for the years 2017, and 2018 as well. Also, the accuracy of this study’s results is causally linked to the accuracy of the service statistics of CMB. Although we applied the triangulation method to provide valid WISN estimates (the review of laboratory records, the interviews with key laboratory personnel and the observation of laboratory processes and time measurements of laboratory activities), the results of this study cannot be generalized to other laboratories of different healthcare levels (primary, secondary) with different quantity of workload, different level of automation and IT. Medical laboratory staffing plans require periodic revision because of changes in the volume, technology and nature of the activities. Running the WISN assessment every 2 years can assist in evaluation of management efforts.

## Conclusion

This study demonstrated the implementation process of the WISN methodology and its usefulness for estimating real staff requirements and activity standards in the high-volume clinical laboratories and translates workload into medical biochemists/laboratory technicians FTEs. It showed critical shortages and inequalities in the distribution of workforce among laboratories. The WISN method can aid the healthcare policymakers to prioritize the recruitment of certain laboratory professionals, optimize utilization of the existing workforce, reallocate them based on the existing workload, develop normative guideline in the field of clinical laboratory diagnostics and deliver quality services. 

## Supplementary Information

**Additional file 1: Figure S1.** Organisational structure of Center for Medical Biochemistry University Clinical Center of Serbia.

**Additional file 2. **WISN steps.

**Additional file 3: Figure S2.** Flowcharts of laboratory activities.

**Additional file 4. **Category and individual allowance standards for medical biochemists/medical laboratory technicians, Center for Medical Biochemistry University Clinical Center of Serbia, 2019.

## Data Availability

All data generated or analysed during this study are included in this published article and its additional information files.

## Abbreviations

AWT: Available working time
CAF: Category allowance factor
CAS: Category allowance standards
CMB UCCS: Center for Medical Biochemistry of the University Clinical Centre of Serbia
CMB: Center for Medical Biochemistry
COVID-19: Coronavirus disease 2019
FTEs: Full-time equivalents
IAF: Individual allowance factor
IAS: Individual allowance standards
ISO: International Organization for Standardization
UCCS: University Clinical Centre of Serbia
WHO: World Health Organization
WISN: Workload Indicators of Staffing Need
