# Supplementary material for: Use of the WISN method to assess the health workforce requirements for the high-volume clinical biochemical laboratories
Source: Hum Resour Health. 2022 Jan 28;19(Suppl 1):143. doi: 10.1186/s12960-021-00686-w (PMC8795329; doi:10.1186/s12960-021-00686-w)
Supplement: Supplementary file 1 — Additional file 1: Figure S1. Organisational structure of Center for Medical Biochemistry University Clinical Center of Serbia. [file 12960_2021_686_MOESM1_ESM.docx]

Additional file 1:

Figure S1. Organisational structure of Center for Medical Biochemistry University Clinical Center of Serbia


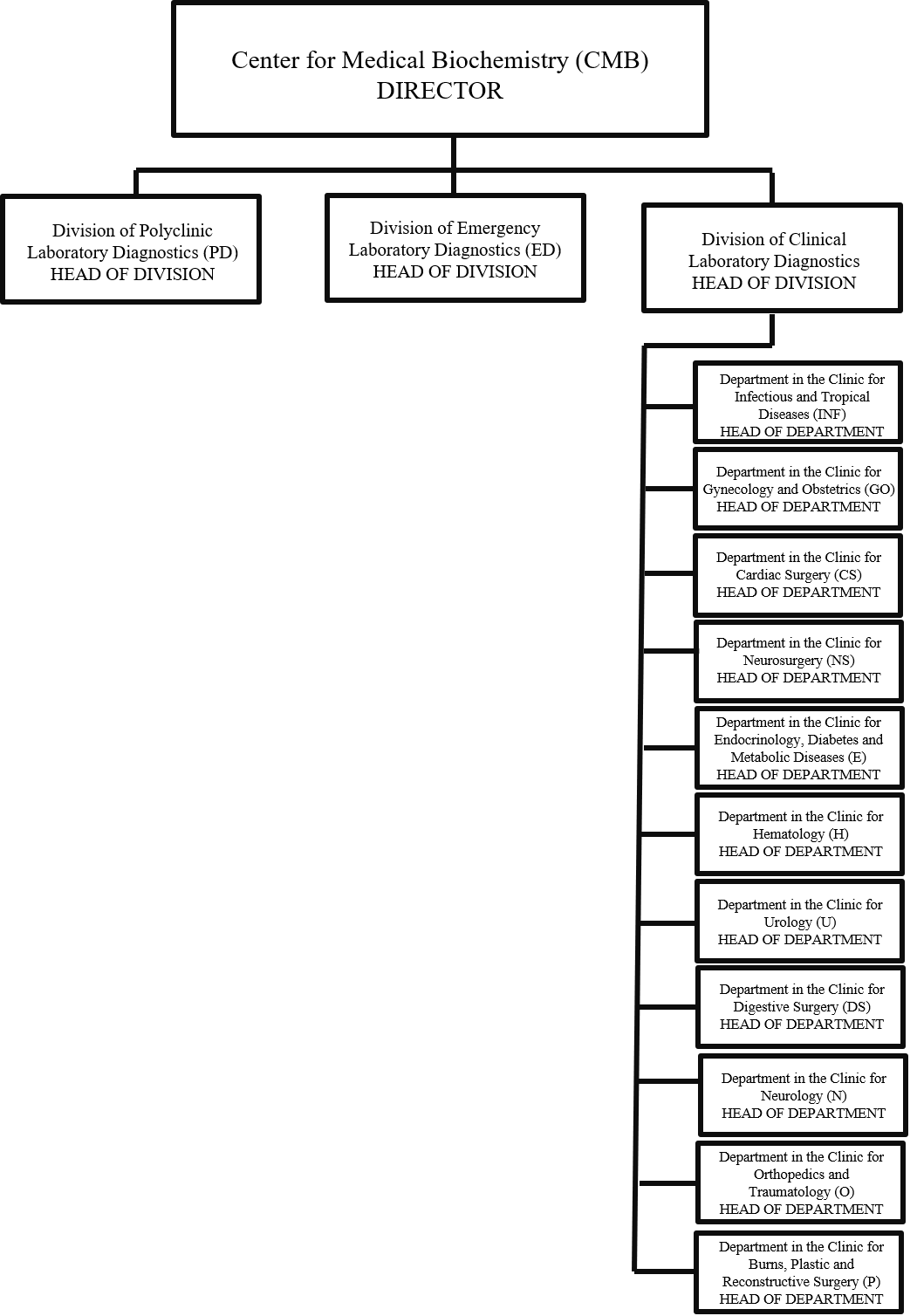


Division of Polyclinic Laboratory Diagnostics (1PD), Division of Emergency Laboratory Diagnostics (2ED), and Division of Clinical Laboratory Diagnostics in Department in the Clinic for Infectious and Tropical Diseases (3INF), Department in the Clinic for Gynaecology and Obstetrics (4GO), Department in the Clinic for Cardiac Surgery (5CS), Department in the Clinic for Neurosurgery (6NS), Department in the Clinic for Endocrinology, Diabetes and Metabolic Diseases (7E), Department in the Clinic for Haematology (8H), Department in the Clinic for Urology (9U), Department in the Clinic for Digestive Surgery (10DS) , Department in the Clinic for Neurology (11N), Department in the Clinic for Orthopaedics and Traumatology (12O) and Department in the Clinic for Burns, Plastic and Reconstructive Surgery (13P).
