# Supplementary material for: Use of the WISN method to assess the health workforce requirements for the high-volume clinical biochemical laboratories
Source: Hum Resour Health. 2022 Jan 28;19(Suppl 1):143. doi: 10.1186/s12960-021-00686-w (PMC8795329; doi:10.1186/s12960-021-00686-w)
Supplement: Supplementary file 2 — Additional file 2. WISN steps. [file 12960_2021_686_MOESM2_ESM.docx]

Additional file 2: WISN Steps

The **first WISN step** determines priority cadre for which staffing assessment is required.

The **second WISN step** calculates the available working time (AWT) that represents the total number of working hours available for each laboratory staff category in one year, considering authorised and unauthorised absences. using the formula: AWT=[A−(B+C+D+E)] * F where A is the number of possible working days in one year, B is the number of public holidays, C is the number of annual leaves (earned, casual leaves, relocation, death in the family, continuing medical education, entitled layoffs etc.), D is the number of days off due to sick leaves in one year, E is the number of days off due to other leaves in one year (training, personal leave, union activity), and F is the number of working hours in 1 one day. Half an hour of rest was considered for each staff category per shift. The number of annual working and nonworking days were obtained from the official government calendar for public sector institutions, and the information about the other absences from work from the laboratory staff’s record of personnel information.

The **third WISN step** define all the current workload components of laboratory cadre: core laboratory service activities (performed by all employees of a specific profile and regular statistics are collected on them), additional categorical activities (performed by all members of the cadre, but regular statistics are not collected on them), and additional individual activities (performed only by certain (not all) members of the cadre and regular statistics are not collected on them). The direct observation of the laboratory working processes and examination of standard operating procedures and documentation of laboratories were also used for that purposes.

The **fourth WISN step** sets activity standards. An activity standard is the time necessary for a well-trained, skilled, motivated worker to perform an activity to professional standards in the local circumstances. Two types of activity standards are defined: service standards as standards for core laboratory service activities and allowance standards as activity standards determined for support activities (category allowance standards (CAS)) and additional activities (individual allowance standards (IAS)). CAS (expressed in %) is calculated as the percentage of AWT spent on each support activity. IAS (expressed in time units) is calculated multiplying the number of laboratory staff members by the time that each additional activity requires in one year. Activity standards were established based on daily records and interview with laboratory staff.

The **fifth WISN step** establishes standard workloads that represents the amount of work that laboratory professional can do in one year within all core laboratory activities according to professional standards. Standard workload is calculated by dividing the AWT in one year by the unit time for the activity.

The **sixth WISN step** is calculating allowance factors. The category allowance factor (CAF) is a multiplier that is used to calculate the total number of full-time equivalent (FTE) medical biochemists/laboratory technicians, required for categorical additional activities CAF = 1 / [1 – (Total CAS / 100)]. The individual allowance factor (IAF) shows how many FTE laboratory staff (or what proportion of such a staff member’s time) are needed to cover additional activities of certain cadre members. IAF is calculated by dividing the total IAS by total AWT.

The **seventh WISN step** determines staff requirements based on WISN. The required number of FTE laboratory workers to accomplish core laboratory service, and additional activities of certain cadre members is calculated by equation WISN = (N * CAF) +IAF, where N is number of laboratory workers need to perform the core laboratory activities.

The **eighth WISN step** is analysing and interpreting WISN results. The WISN results could be analysed considering the difference between current and required staff number (difference shows surplus or shortage of laboratory workers) or the WISN ratio of the current and the required number of laboratory workers (WISN ratio of 1.0 is an indication that staffing and workload are in balance; WISN ratio greater than 1 is evidence of overstaffing in relation to the workload (low workload pressure); WISN ratio less than 1 indicates that the current staffing level is insufficient to overcome a certain workload (moderate or high pressure).
