# Supplementary material for: Use of the WISN method to assess the health workforce requirements for the high-volume clinical biochemical laboratories
Source: Hum Resour Health. 2022 Jan 28;19(Suppl 1):143. doi: 10.1186/s12960-021-00686-w (PMC8795329; doi:10.1186/s12960-021-00686-w)
Supplement: Supplementary file 3 — Additional file 3: Figure S2. Flowcharts of laboratory activities. [file 12960_2021_686_MOESM3_ESM.docx]

Additional File 3:

Figure S2. Flowcharts of laboratory activities

Figure S2A. Process of laboratory activities in laboratories without laboratory information system, Center for Medical Biochemistry University Clinical Center of Serbia, 2019

Figure S2B. Process of laboratory activities in laboratories with laboratory information system, Center for Medical Biochemistry University Clinical Center of Serbia, 2019

Figure S2C. Process of laboratory activities in laboratories with laboratory information system and preanalytics, Center for Medical Biochemistry University Clinical Center of Serbia, 2019

Figure S2D. Process of laboratory activities in laboratories with laboratory information system and total laboratory automation with storage, Center for Medical Biochemistry University Clinical Center of Serbia, 2019

Start

Finish

Sample reception and assignement of laboratory number

Laboratory technician at the reception counter

Entering data from the laboratory instruction into the laboratory protocol

Laboratory technician in the administrative part of the laboratory

Pre-analytical

phase

Samples triage

Laboratory technician in the sample triage section

Preanalytical sample preparation (putting samples on centrifuge, removing from centrifuge de-capping, aliquoting, assessment of interferences preparation, transfer to other analyzers or to other laboratories)

Laboratory technician in the sample triage section

Laboratory .technician in the analysing section

Loading tubes on analyzer

Analytical

phase

Non-standard fluids analysis

Laboratory technician in the analyzing section

Analyzing samples

Laboratory technician in the analyzing section

Removing samples from analyzer on analyzers

Sample tube capping and

putting tubes in the refrigerator

Laboratory technician in the analyzing section

Laboratory technician in the administrative part of the laboratory

Entering test results into the laboratory protocol and laboratory report

Post-analytical

phase

Verification and issuing of laboratory report

Responsible medical biochemist

Laboratory technician

Distribution of laboratory reports in the registers of CCS services

Laboratory technician

Disposal of expired samples

Figure S2A. Process of laboratory activities in laboratories without laboratory information system, Center for Medical Biochemistry University Clinical Center of Serbia, 2019

Start

Sample reception

Laboratory technician at the reception counter

Entering data from the laboratory instruction to laboratory information system, barcode print, sample labeling

barcode print

system

Laboratory technician in the administrative part of the laboratory

Pre-analytical

phase

Laboratory technician in the sample triage section

Samples triage

Preanalytical sample preparation

(putting samples on centrifuge, removing from centrifuge de-capping, aliquoting, assessment of interferences preparation, transfer to other analyzers or to other laboratories)

Laboratory technician in the sample triage section

Non-standard fluids analysis

Laboratory technician in the analyzing section

Loading tubes on analyzer

Laboratory technician in the analyzing section

Analytical

phase

Analyzing samples

on analyzers

Laboratory technician in the analyzing section

Removing samples from analyzer on analyzers

Sample tube recapping and

putting tubesin the refrigerator

Laboratory technician in the analyzing section

Responsible medical biochemist

Verification of results in laboratory

information system

Post-analytical

phase

Issuing of laboratory report

Responsible medical biochemist

Laboratory technician

Distribution of laboratory reports in the registers of CCS services

Laboratory technician

Disposal of expired samples

Finish

Figure S2B. Process of laboratory activities in laboratories with laboratory information system, Center for Medical Biochemistry University Clinical Center of Serbia, 2019

Start

Laboratory technician at the reception counter

Sample reception

Entering data from the laboratory instruction to laboratory information system, barcode print, sample labeling

barcode print

system

Laboratory technician in the administrative part of the laboratory

laboratory

Receiving samples from outpatients clinics, clinics that do not have a laboratory and other CMB laboratories

Pre-analytical

phase

Laboratory technician in the triage section “small” triage

“amall

Laboratory technician in the sample triage section

Samples triage

Samples decapping

Laboratory technician in the analysing section

Putting samples on pre-analytical system

Non-standard fluids analysis

Laboratory technician in the analysing section

Analytical

phase

Automatic centrifugation and analysis

Laboratory technician in the analysing section

Removing samples from analyzer

Laboratory technician in the analyzing section

Sample tube recapping and

putting tubes

in the refrigerator

Responsible medical biochemist

Verification of results in laboratory

information system

Post-analytical

phase

Responsible medical biochemist

Issuing of

laboratory report

Distribution of laboratory reports in the registers of CCS services

Laboratory technician

Laboratory technician

Disposal of expired samples

Finish

Figure S2C. Process of laboratory activities in laboratories with laboratory information system and preanalytics, Center for Medical Biochemistry University Clinical Center of Serbia, 2019

Start

Admission and registration of patients, barcode print

(unos podataka u LIS)

Sample reception (morning urine, 24h urine,feces, etc.)

Laboratory technician at the reception counter

Patient admission

and

bood sampling

samples labeling

Laboratory technician in the sampling booth

Pre-analytical

phase

Laboratory technician in the triage section “small” triage

Laboratory technician in the biological material reception section

Entering data from the laboratory instruction to laboratory information system, barcode print, sample labeling

barcode print

system

Receiving samples from outpatients clinics, clinics that do not have a laboratory and other CMB laboratories

Laboratory technician in the sample triage section

Samples

triage

Laboratory technician in the analysing section

Putting samples on pre-analytical system

Laboratory technician in the analyzing section

Non-standard fluids analysis Electrophoresis

Automatic centrifugation, de-capping, analysis, recapping, automatic archiving storage racks, automatic samples disposal.

Analytical

phase

Responsible medical biochemist

Verification of results in laboratory

information system

Post-analytical

phase

Responsible medical biochemist

Issuing of

laboratory report

Distribution of laboratory reports in the registers of CCS services

Laboratory technician

Laboratory technician

Disposal of expired samples

Finish

Figure S2D. Process of laboratory activities in laboratories with laboratory information system and total laboratory automation with storage, Center for Medical Biochemistry University Clinical Center of Serbia, 2019
