# Supplementary material for: Use of the WISN method to assess the health workforce requirements for the high-volume clinical biochemical laboratories
Source: Hum Resour Health. 2022 Jan 28;19(Suppl 1):143. doi: 10.1186/s12960-021-00686-w (PMC8795329; doi:10.1186/s12960-021-00686-w)
Supplement: Supplementary file 4 — Additional file 4. Category and individual allowance standards for medical biochemists/medical laboratory technicians, Center for Medical Biochemistry University Clinical Center of Serbia, 2019. [file 12960_2021_686_MOESM4_ESM.docx]

Additional File 4: Category and individual allowance standards for medical biochemists/medical laboratory technicians, Center for Medical Biochemistry University Clinical Center of Serbia, 2019

Table S1A. Category allowance standards for medical biochemists, Center for Medical Biochemistry University Clinical Center of Serbia, 2019

Table S1B. Category allowance standards for medical laboratory technicians, Center for Medical Biochemistry University Clinical Center of Serbia, 2019

Table S2A Individual allowance standards for medical biochemists, Center for Medical Biochemistry University Clinical Center of Serbia, 2019

Table S2B. Individual allowance standards for medical laboratory technicians, Center for Medical Biochemistry University Clinical Center of Serbia, 2019

Table S1A. Category allowance standards (CAS) for medical biochemists, Center for Medical Biochemistry University Clinical Center of Serbia, 2019

| **Staff category: Medical biochemists** | | |
| --- | --- | --- |
| **Workload**  **group** | **Workload**  **components** | **Category allowance standards (CAS)**  **(actual working time)** |
| Support  activities | Workplace preparation (preparing analyzer for operation, reviewing calibration/QC analysis results) | 5 minutes per day |
|  | Internal communication/consultation (with physicians, nurses by phone and in person) | 30 minutes per day |
|  | Staff supervision meetings | 8 days per year |
|  | Continuing medical education | 9 days per year |

Table S1B. Category allowance standards (CAS) for medical laboratory technicians, Center for Medical Biochemistry University Clinical Center of Serbia, 2019

| **Staff category: Medical laboratory technicians** | | |
| --- | --- | --- |
| **Workload**  **group** | **Workload**  **components** | **Category allowance standards (CAS)**  **(actual working time)** |
| Support  activities | Workplace preparation and maintenance (Analyzer maintenance, and preparing analyzer for operation - calibration/internal QC; environmental/refrigeration temperature monitoring, controlling and recording, maintaining a safe, tidy environment) | 50 minutes per day |
|  | Internal communication/consultation (with physicians, nurses by phone and in person) | 30 minutes per day |
|  | Staff supervision meetings | 8 days per year |
|  | Continuing medical education | 6 days per year |

Table S2A Individual allowance standards (IAS) for medical biochemists, Center for Medical Biochemistry University Clinical Center of Serbia, 2019

| **Staff category: Medical biochemists** | | | | | | | |
| --- | --- | --- | --- | --- | --- | --- | --- |
| **Workload**  **group** | **Workload**  **components** | **Individual additional standards (IAS)**  **per type of laboratory and number of FTE** | | | | | |
|  |  | n | PD lab | n | ED lab | n | Other labs |
| Additional  activities | Quality management system - commission (peer evaluations or assessments; drafting, revising and reading standard operating procedures; laboratory accreditation, participation in inter-laboratory comparisons (i.e., ILC) and/or external proficiency testing (i.e., PT) schemes) | 9  1 | 3 days per month  5 days per month   \|  \| \| --- \| \|  \| \|  \| | 9  2 | 3 days per month  4 days per month | 1  1 | 8 days per year  2 days per month   \|  \| \| --- \| \|  \| |
|  | Documentation preparation (analyzer operating instructions, methods validation, measurement uncertainty determination, writing notices) | 5 | 3 days per year | 5 | 3 days per year | 1 | 3 days per year |
|  | Planning (education, UCCS integrated work plan, laboratory improvement  plan, and monitoring the implementation) | 4  2 | 2 days per year  6 days per year | 4 | 2 days per year | 1 | 2 days per year |
|  | Procurement planning & Reporting (laboratory level supply & stock checking reagent and consumables) | 4  4 | 30 minutes per month  2 day per month | 4  4 | 30 minutes per month  2 day per month | 1  1 | 30 minutes per month  2 day per month |
|  | Teaching/In-Service (students, professionals, on-the job education) | 9  1 | 30 minutes per day  4 days/year | 9  1 | 30 minutes per day  4 days/year | 1 (2) | 30 minutes per day |
|  | External meetings (Board/Committee Functions, public relations, participation in professional association committees, etc.) | 3  5  1 | 1 day per month  1.5 hours per month  2 days per month | 3  5 | 1 day per month  1.5 hours per month | 1  1 | 1 day per month  1.5 hours per month |
|  | Commercial/non-commercial clinical research | 1  3 | 3 day per year  30 minutes per month | 13 | 3 days per year  30 minutes per month | 1 | 30 minutes per month |
|  | Surveys for internal purposes | 2 | 3 days per year | 2 | 3 days per year | 1 | 3 days per year |

n-number of FTE medical laboratory technicians

PD - Division of Polyclinic Laboratory Diagnostics

ED - Division of Emergency Laboratory Diagnostics

Other labs - Division of Clinical Laboratory Diagnostics in Department in the Clinic for Infectious and Tropical Diseases, Department in the Clinic for Gynecology and Obstetrics, Department in the Clinic for Cardiac Surgery, Department in the Clinic for Neurosurgery, Department in the Clinic for Endocrinology, Diabetes and Metabolic Diseases, Department in the Clinic for Hematology, Department in the Clinic for Urology, Department in the Clinic for Digestive Surgery, Department in the Clinic for Neurology, Department in the Clinic for Orthopedics and Traumatology, Department in the Clinic for Burns, Plastic and Reconstructive Surgery.

Table S2B. Individual allowance standards for medical laboratory technicians, Center for Medical Biochemistry University Clinical Center of Serbia, 2019

| **Staff category: Medical laboratory technicians** | | | | | | | |
| --- | --- | --- | --- | --- | --- | --- | --- |
| **Workload**  **group** | **Workload**  **components** | **Individual additional standards (IAS)**  **per type of laboratory and number of FTE** | | | | | |
|  |  | n | PD lab | n | ED lab | n | Other labs |
| Additional  activities | Quality management (quality management meetings; reading standard operating procedures; laboratory accreditation, participation in inter-laboratory comparisons (ie., ILC) and/or external proficiency testing (ie., PT) schemes) | 2  3 | 2 hours per month  4 hours per year | 23 | 2 hours per month  4 hours per year | 1  1.5* | 2 hours per month  1 hour per year |
|  | Creating work schedules | 1 | 1 day per month | 1 | 1 day per month | 1 | 1 day per month |
|  | Reagents and consumable reception/storage/inventory records | 2  3 | 6,7 hours per day  2 days per month | 3 | 2 days per month | 1 | 2 days per month |
|  | Reporting  (recording and calculating workload and other statistical data, preparing consumption reports, recording of worthless registry material etc.) | 2 | 4 days per month | 2 | 4 days per month | 1 | 4 days per month |
|  | Teaching/In-Service  (technicians on practice, in-service education) | 5 | 30 minutes per day | 5 | 30 minutes per day | 1.5* | 30 minutes per day |
|  | Employee Meetings | 5 | 45 minutes per month | 5 | 30 minutes per month | 1 | 30 minutes per month |
|  | Surveying (patients and/or clinicians, employees) for internal purposes | 1  1 | 1 day per year  30 minutes per year | 1 | 1 hour per year | 1 | 30 minutes per year |

n-number of FTE medical laboratory technicians

PD - Division of Polyclinic Laboratory Diagnostics

ED - Division of Emergency Laboratory Diagnostics

Other labs - Division of Clinical Laboratory Diagnostics in Department in the Clinic for Infectious and Tropical Diseases, Department in the Clinic for Gynecology and Obstetrics, Department in the Clinic for Cardiac Surgery, Department in the Clinic for Neurosurgery, Department in the Clinic for Endocrinology, Diabetes and Metabolic Diseases, Department in the Clinic for Hematology, Department in the Clinic for Urology, Department in the Clinic for Digestive Surgery, Department in the Clinic for Neurology, Department in the Clinic for Orthopedics and Traumatology, Department in the Clinic for Burns, Plastic and Reconstructive Surgery.

*1.5 refers to the situation that this is a duty of one person in some laboratories while in others, large, laboratories, 2 persons perform this activity.
